# Emerging Multifunctional Materials for Next-Generation Energy Systems

**DOI:** 10.3390/molecules31142508

**Published:** 2026-07-17

**Authors:** Zhao Ding, Shicong Yang

**Affiliations:** 1 College of Materials Science and Engineering, National Engineering Research Center for Magnesium Alloys, Chongqing University, Chongqing 400044, China; 2 Faculty of Metallurgical and Energy Engineering, Kunming University of Science and Technology, Kunming 650093, China; shicongyang@kust.edu.cn

The accelerating transition toward low-carbon and resource-efficient energy systems is redefining the performance requirements imposed on functional materials [1,2,3,4]. Future materials must do more than deliver a single optimized property: they are expected to integrate efficient energy conversion or storage with interfacial stability, environmental compatibility, mechanical reliability, and scalable manufacturing [5,6,7,8]. Based on this background, the Special Issue “Emerging Multifunctional Materials for Next-Generation Energy Systems” comprises a diverse collection of studies demonstrating how multiple functions can be deliberately coupled through composition, architecture, interface design, and processing.

The ten papers within this Special Issue collectively illustrate the breadth of multifunctional materials research across the energy–environment nexus. These contributions can be systematically organized into four complementary research directions: (1) circular resource valorization and environmental remediation, (2) interfacial engineering for energy conversion and functional protection, (3) next-generation electrochemical energy storage and safety, and (4) advanced characterization and evidence reliability. Rather than representing isolated technological topics, these directions form a connected research chain extending from sustainable feedstock utilization and functional-material construction to device-level reliability and the validation of underlying mechanisms.

Circular resource utilization constitutes the first major theme of this collection. Xiong et al. (Contribution 1) provided a systematic review of silicon recovery from diamond-wire sawing silicon powder waste, classifying existing hydrometallurgical processes into direct acid leaching, pyrometallurgical treatment followed by acid leaching, and acid leaching followed by pyrometallurgical purification. Their analysis identified the latter route as a particularly effective pathway for achieving high-purity silicon recovery, while also highlighting emerging opportunities involving external field-assisted purification, oxygen-regulated leaching, and surfactant-assisted organic-acid treatment. Complementing this recovery-oriented perspective, Li et al. (Contribution 2) demonstrated that the same class of photovoltaic silicon waste can serve as a reactive feedstock for hydrogen production. Through short-duration vibration grinding with KCl, the authors obtained a hydrogen yield of 86.1% and an initial hydrogen generation rate of 399.37 mL min^−1^ (g DSSW)^−1^. At 338 K, approximately 85% conversion was achieved within 100 s, accompanied by an initial hydrogen generation rate of 1383.6 mL min^−1^ (g DSSW)^−1^. Together, these two studies reveal two complementary valorization routes for silicon waste: purification and reintegration into the photovoltaic material cycle, or direct chemical conversion into a clean energy carrier.

The concept of waste-derived multifunctionality is further extended from photovoltaic residues to biomass and industrial byproducts. Kan et al. (Contribution 3) converted coffee shells into efficient and regenerable adsorbents for Rhodamine B removal. High-temperature pyrolysis promoted pore development and aromaticity, while subsequent water washing removed inorganic ash and exposed additional active sites. The optimized WCB700 biochar exhibited a specific surface area of 273.6 m^2^ g^−1^ and an adsorption capacity of 193.5 mg g^−1^, while retaining more than 85% of its initial capacity after five regeneration cycles. The adsorption behavior was attributed to the combined effects of pore filling, π–π interactions, hydrogen bonding, and electrostatic attraction. Zhu and Wei (Contribution 5) similarly transformed industrial silica fume into a functional adsorbent through direct surface activation and molecular grafting. The resulting TACA-APTES-functionalized silica fume achieved a maximum Cd^2+^ adsorption capacity of 91.37 mg g^−1^ and maintained substantial removal efficiency over five reuse cycles. These studies demonstrate that waste valorization is not merely a disposal strategy; through controlled thermal processing and surface chemistry, secondary resources can become high-value functional materials for environmental protection and may indirectly improve the sustainability of energy-related industries.

A second group of contributions highlights interfacial engineering as a common design principle connecting electrocatalysis, electromagnetic-wave management, and photovoltaics. Liu et al. (Contribution 4) reviewed recent synthesis strategies and mechanistic understanding of Co_3_O_4_-based oxygen evolution electrocatalysts. Their analysis links synthesis routes to particle size, morphology, elemental composition, coordinatively unsaturated sites, electronic interactions, and surface reconstruction under operating conditions. Importantly, this contribution shows that catalytic performance cannot be understood solely from the initial crystal structure; the dynamically reconstructed surface and its local coordination environment frequently determine the actual reaction pathway and active state.

Zhang et al. (Contribution 7) extended hierarchical and interfacial design into the field of electromagnetic-wave absorption. By integrating a biomass-derived porous carbon framework with magnetic phases, the authors constructed a multiscale architecture that combined conductive loss, interfacial polarization, magnetic resonance, and multiple scattering. The optimized BPC-0.9 composite achieved a minimum reflection loss of −45.17 dB at a thickness of only 1.8 mm, while BPC-0.6 delivered an effective absorption bandwidth of 6.65 GHz at 2.2 mm. Such materials are relevant not only to electromagnetic-pollution mitigation but also to the protection and reliable operation of increasingly integrated electronic and energy systems. Liu et al. (Contribution 10) brought the same interface-centered reasoning to Cu_2_ZnSn(S,Se)_4_ solar cells. Their review identifies band misalignment, interface defects, and associated non-radiative recombination as major contributors to the persistent open-circuit-voltage deficit in CZTSSe devices, and systematically examines interface modification and passivation strategies. Collectively, these three contributions establish a coherent principle: effective interfaces must promote desired charge, mass, or energy-transfer processes while suppressing parasitic recombination, impedance mismatch, and dissipative pathways that do not contribute to useful device output.

The third research direction concerns electrochemical energy storage under coupled chemical, structural, and mechanical constraints. He et al. (Contribution 6) systematically investigated the state-of-charge-dependent deformation and electrochemical evolution of sodium-ion batteries subjected to mechanical compression. By correlating structural deformation, voltage and thermal responses, electrochemical impedance, and internal damage, their study showed that ohmic behavior was primarily governed by compression-induced deformation, whereas interfacial and diffusion resistances were jointly affected by deformation and state of charge. This work moves beyond conventional capacity-centered evaluation and demonstrates that mechanical integrity must be treated as an intrinsic component of electrochemical performance and safety.

At the materials level, Xiong et al. (Contribution 9) presented a comprehensive review of manganese-based oxide cathodes for aqueous magnesium-ion batteries. The authors summarized their crystal structures, electrochemical characteristics, optimization strategies, and electrode-reaction mechanisms while also discussing the continuing uncertainty regarding Mg^2+^ insertion, proton participation, and possible co-insertion processes. Particular attention was given to practical limitations including low active-material loading, insufficient areal capacity, and the limited validation of high-capacity full or pouch cells. When considered together, Contributions 6 and 9 emphasize that next-generation batteries require simultaneous optimization across multiple length scales: ion-storage chemistry at the atomic level, electrode architecture at the mesoscale, and mechanical and safety performance at the cell level.

The final contribution provides a methodological foundation for interpreting many of the materials and interfaces discussed above. Chen et al. (Contribution 8) critically examined focused-ion-beam artifacts and evidence reliability in advanced microscopy of energy materials. Focused-ion-beam scanning electron microscopy enables site-specific access to buried interfaces, particle interiors, porous electrodes, and localized degradation regions that are otherwise difficult to examine. However, ion-beam-induced amorphization, implantation, redeposition, curtaining, local heating, roughening, and phase mixing can alter precisely the structures that the analysis aims to reveal. The authors therefore proposed that microscopy evidence should be assessed according to the scientific claim being made and advocated artifact-aware preparation, low-energy finishing, cryogenic or alternative-ion strategies where appropriate, and validation using orthogonal characterization methods. This perspective completes the research chain represented by the Special Issue: multifunctional properties must not only be designed and measured, but must also be supported by evidence whose preparation history and limitations are explicitly understood.

Taken together, the papers in this Special Issue demonstrate that multifunctionality should not be interpreted as the simple accumulation of several favorable properties in a single material. More fundamentally, it involves the deliberate coupling of functions across the material–interface–device–system hierarchy. Waste-derived feedstocks must be compatible with purification, functionalization, and scalable processing; catalytic and photovoltaic interfaces must accelerate desired transfer processes while suppressing competing losses; electrochemical storage materials must reconcile ion transport with structural and mechanical stability; and mechanistic conclusions must be supported by artifact-aware and claim-specific evidence.

This collection has highlighted several priorities for future research. Circularity should be incorporated at the initial stage of materials design rather than evaluated only after device fabrication. Interface engineering must progress from empirical modification toward quantitative control of local coordination, charge redistribution, reaction intermediates, and dynamically reconstructed states. Electrochemical evaluation should more closely reproduce realistic mass loading, mechanical constraint, and cell-level operating conditions. Meanwhile, in situ and operando characterization, low-damage microscopy, multiscale modeling, high-throughput experimentation, and artificial intelligence-assisted materials discovery are expected to shorten the path from structural hypothesis to validated mechanism. The integration of these approaches with life-cycle assessment, cost analysis, and scalable manufacturing will ultimately determine whether emerging multifunctional materials can move from laboratory demonstrations to resilient next-generation energy systems.

## References

[1] Chu S., Majumdar A. (2012). Opportunities and challenges for a sustainable energy future. Nature.

[2] Aricò A.S., Bruce P., Scrosati B., Tarascon J.-M., van Schalkwijk W. (2005). Nanostructured materials for advanced energy conversion and storage devices. Nat. Mater..

[3] Lewis N.S. (2016). Research opportunities to advance solar energy utilization. Science.

[4] Grey C.P., Tarascon J.-M. (2017). Sustainability and in situ monitoring in battery development. Nat. Mater..

[5] Geissdoerfer M., Savaget P., Bocken N.M.P., Hultink E.J. (2017). The Circular Economy—A new sustainability paradigm?. J. Clean. Prod..

[6] Larcher D., Tarascon J.-M. (2015). Towards greener and more sustainable batteries for electrical energy storage. Nat. Chem..

[7] Ding Z., Li Y., Yang H., Lu Y., Tan J., Li J., Li Q., Chen Y., Shaw L.L., Pan F. (2022). Tailoring MgH_2_ for hydrogen storage through nanoengineering and catalysis. J. Magnes. Alloys.

[8] Garcia-Navarro J., Isaacs M.A., Favaro M., Ren D., Ong W., Grätzel M., Jiménez-Calvo P. (2024). Updates on hydrogen value chain: A strategic roadmap. Glob. Chall..

